# Short-term transport stress and supplementation alter immune function in aged horses

**DOI:** 10.1371/journal.pone.0254139

**Published:** 2021-08-19

**Authors:** Ashton B. Miller, Patricia A. Harris, Virginia D. Barker, Amanda A. Adams

**Affiliations:** 1 Department of Veterinary Science, M.H. Gluck Equine Research Center, University of Kentucky, Lexington, Kentucky, United States of America; 2 Waltham Petcare Science Institute, Waltham-on-the-Wolds, Leicestershire, England, United Kingdom; Lewis Katz School of Medicine, Temple University, UNITED STATES

## Abstract

Long-distance transport is associated with stress-related changes in equine immune function, and shipping-associated illnesses are often reported. Horses are frequently transported short distances, yet the effects of short-term transport on immune function remain largely unknown. Twelve horses, aged 15–30 yr, were assigned to either the control (n = 6) or treatment (n = 6) groups; treatment horses received a daily antioxidant supplement 3 weeks before and after transport. All horses were transported for approximately 1.5–2 hr on Day 0. Blood was collected via jugular venipuncture at 15-min pre- and post-transport and on Days –21, 1, 3, 7, 14, and 21. Body temperature, heart rate, body weight, total cortisol, and gene expression of IFNγ, IL-1β, IL-2, IL-4, IL-6, IL-8, IL-10, IL-12α, IL-17α, SAA1, and TNFα in whole blood were measured. Peripheral blood mononuclear cells were isolated, stimulated with PMA/ionomycin, and stained for IFNγ and TNFα before analysis via flow cytometry. Statistical analyses were performed with significance set at *P* < 0.05 (SAS 9.4). Transport and supplementation did not appear to affect body weight, heart rate, IL-4, IL-8, IL-12α, IL-17α, change (Δ) in the % and mean fluorescence intensity (MFI) of IFNγ^+^ lymphocytes after stimulation, or Δ in the % and MFI of TNFα^+^ lymphocytes after stimulation. Supplementation decreased IL-1β and SAA1 expression. Transport increased total cortisol concentration, body temperature, and IL-2, IL-6, and IL-10 expression but decreased IL-1β, TNFα, and IFNγ expression. Short-term transportation affected physiological, endocrine, and immune responses; supplementation may ameliorate inflammation in aged horses. Immune responses were most altered at 15-min post-transport and typically recovered by Day 1, suggesting that horses may be vulnerable to disease during and almost immediately after short-term transport.

## Introduction

Horses are frequently transported for sporting and breeding purposes. Often, horse owners and veterinarians report “shipping fever” or pleuropneumonia after horses are transported [1–5]. These cases of pleuropneumonia are generally of bacterial etiology, and *Streptococcus equi* spp. *zooepidemicus*, a commensal bacterium of the equine lung, is frequently isolated [2,5–8]. Studies have shown that altering conditions within trailers, e.g., restraint length, head position, and water/hay access, can reduce the risk of transport-related illness [2,5,7,9,10]. However, management changes do not eliminate the risk entirely, indicating that immune function is altered because of transport, and thus, bacteria, such as *S*. *zooepidemicus*, can establish and induce pleuropneumonia. Alterations in immune function can further compromise the tissue, which may allow for greater risk of viral respiratory illness as well [11]. Because horses are often shipped in the company of other horses and tend to then reside in new stable environments, it is concerning that immune function may be compromised during transport. One recent study showed that approximately half of 167 horses that had recently undergone lengthy travel while being imported to the U.S. from Europe were positive for equine herpesvirus (EHV) 1, 2, 4, and/or 5 [12]. This further suggests that transport-induced alterations in immune function allow for recrudescence of latent viral infection and potential transmission to other horses in new locations, and it is particularly concerning.

While long-term transportation (i.e., ≥ 24 hr duration) is known to affect immune function in horses and other species, the extent to which short-term transport (i.e., ≤ 8 hr duration) impacts both local and systemic immune function remains unclear, although similar physiological and endocrine factors are affected regardless of transport length [9,13–17]. Additionally, many studies have involved transporting horses to new environments, which makes it more difficult to isolate the effects of transport versus the effects of moving horses to new locations with new exposures [18]. Horse owners often transport their horses locally and return home within the same day, which makes it important to understand whether shorter trips also impact immune function. Therefore, the first objective of this study was to examine the effects of short-term transport (less than 2 hr) on immune function in aged horses who were returned home afterwards. The second objective was to investigate whether an antioxidant supplement formulated to help support airway function would enhance immune function overall and confer protective responses during and after transport [19,20].

## Materials and methods

### Animals

Twelve university-owned research horses between 15 and 30 yr of age were selected. The control group (age range: 15–30 yr; median age: 24 yr) and the treatment group (age range: 16–30 yr; median age: 25.5 yr) each included 6 horses. There was no difference in age between the groups (*P* = 0.8335). The groups consisted of a total of 7 mares and 5 geldings of various breeds, weighing 419.573–620.515 kg (mean ± SD: 528.927 ± 55.004 kg). All horses were housed on the same farm and were primarily housed on pasture. At the time of the study, all of the horses were retired and were sometimes transported by trailer on and/or around the research farm (i.e., short duration/distance). The horses had varying levels of transport experience, and approximately half of the horses likely had significant transport experience at earlier points in their lives. All procedures were approved by the University of Kentucky’s Institutional Animal Care and Use Committee (Protocol #: 2018–3058).

### Study design

Beginning on Day –35, all horses were acclimated to eating their grain and/or pellet rations in individual stall-size pens, which were placed in or next to the horses’ pastures. Blood was also collected via jugular venipuncture on Day –35 for the analysis of interferon gamma (IFNγ)^+^ and tumor necrosis factor alpha (TNFα)^+^ lymphocytes in isolated peripheral blood mononuclear cells (PBMCs) via flow cytometry. Horses were then assigned to control and treatment groups based on these results, so that the groups were initially matched for age and cytokine production by lymphocytes on Day –35. Beginning on Day –21 and continuing for the duration of the study, the treatment group received a daily antioxidant supplement (Winergy Ventil–ate^®^, MARS Horsecare UK, Milton Keynes, UK). The supplement was fed based on body weight and according to the supplement’s label recommendation [100g daily (2 measures) for horses weighing between 450 kg and 600 kg]. Because all of the treated horses fell within this weight range on Day –21 (weighing 453.593–591.485 kg), they each received 100g (2 measures) daily.

Horses were transported in multiple sets over 2 days. On each day, an equal number of control and treatment horses were transported in a 4-horse, slant-load trailer. The same driving route was taken for each trip, although the total travel time varied between approximately 1.5 and 2 hr due to differences in traffic. All horses were returned to their normal pastures after transportation. Blood was collected on Day –21, 15-min pre- and post-transport (Day 0), and Days 1, 3, 7, 14, and 21 post-transport for examination of serum total cortisol concentration, resting whole blood gene expression, and the percentage and mean fluorescence intensity (MFI) of IFNγ^+^ and TNFα^+^ lymphocytes in isolated PBMCs. Rectal temperature was also measured with a digital thermometer at all of the above time points. Heart rate was assessed at 15-min pre- and post-transport only. Body weight was measured on Day –21 and on four other occasions during the study. Because horses were transported over a 2-day period, each horse’s samples were taken based on their travel day (Day 0), with the exception of the baseline samples (Day –21), and all horses’ samples for each time point were analyzed together. For the baseline samples, all collections were performed on the same day; thus, the baseline samples are either Day –21 or Day –22 depending on the travel day (Day 0). Baseline collections are hereafter referred to as Day –21 samples.

### Sample collection and processing

#### Serum total cortisol

Serum was separated by centrifugation and frozen at –20°C. The serum samples were then sent to Cornell University’s Animal Health Diagnostic Center for analysis of total cortisol by chemiluminescent assay [21].

#### Gene expression in whole blood

Whole blood was collected directly into Tempus^™^ Blood RNA tubes at all eight time points and frozen at –20°C until processed according to the manufacturer’s recommendations. Isolated RNA was reverse-transcribed, and gene expression of IFNγ, interleukin (IL)-1β, IL-2, IL-4, IL-6, IL-8, IL-10, IL-12α, IL-17α, serum amyloid A (SAA) 1, and TNFα was analyzed via RT-PCR as previously described [21–24]. Relative quantities (RQs) were calculated using the 2^-ΔΔCt^ method, and the average of all horses’ Day –21 ΔCt values was used as the calibrator for each respective measure [24,25]. RQ values were natural log (Ln) transformed prior to conducting the statistical analyses.

#### PBMCs: Flow cytometry

At all eight time points, PBMCs were isolated from heparinized blood using a Ficoll density gradient (GE Healthcare) and initially frozen at –80°C before being stored in liquid nitrogen [22]. After the study concluded, cells were thawed, counted, added to 2 wells of a cell culture plate, and incubated at 37°C, 5% CO_2_ for 24 hr. During the last 4 hr of incubation, one well was stimulated with PMA/ionomycin, and all wells received Brefeldin A [22]. Then, cells were transferred to a 96-well plate, fixed in 2% paraformaldehyde, and later stained for analysis of IFNγ^+^ and TNFα^+^ cells using flow cytometry (Attune, ThermoFisher) as previously described; a gate was placed around the lymphocyte population, and the percentage of gated cells and MFI (geometric mean) were analyzed [22,23,26].

### Statistical analyses

All analyses were performed using SAS 9.4 (Cary, NC) with significance set at *P* < 0.05, and results were graphed using GraphPad Prism 9.1.1 (San Diego, CA). The primary comparisons of interest were between the control and treated groups and between time points (effects of transport). Distributions were assessed for gross violations of normality (PROC UNIVARIATE). Horse age at Day –21 was assessed using a pooled t-test (PROC TTEST). Descriptive statistics for body weight at Day –21 were also evaluated (PROC UNIVARIATE).

For all analyses with repeated measures, a linear mixed model (PROC MIXED) was used to analyze the group, time, and group-by-time effects. Either an UN, AR (1), ARMA (1,1), TOEP, or TOEPH variance-covariance structure was selected based on model fit (determined by Bayesian information criterion and Akaike information criterion). If no significant group-by-time effects were present, the analyses were then run with only group and time as main effects. For time effects, significant differences were of interest only when there were differences from baseline (Day –21) and/or when the 15-min post-transport time point was different from most of the other time points. When baseline (Day –21) differences existed between the control and treated groups (*P* < 0.1), the analyses were performed again with baseline values included as a covariate in the model. Pearson correlation coefficients were examined for age and all measures at 15-min post-transport only (PROC CORR).

For analysis of the PBMC flow cytometry data, the stimulated samples (PMA/ionomycin) were analyzed as described earlier (PROC MIXED) using change score methodology. The change (Δ) between the media (unstimulated) and stimulated samples was calculated and analyzed with the media values included as a covariate; these results are reported with the Δ symbol [21].

## Results

### Body temperature, heart rate, and body weight

**Fig 1:**
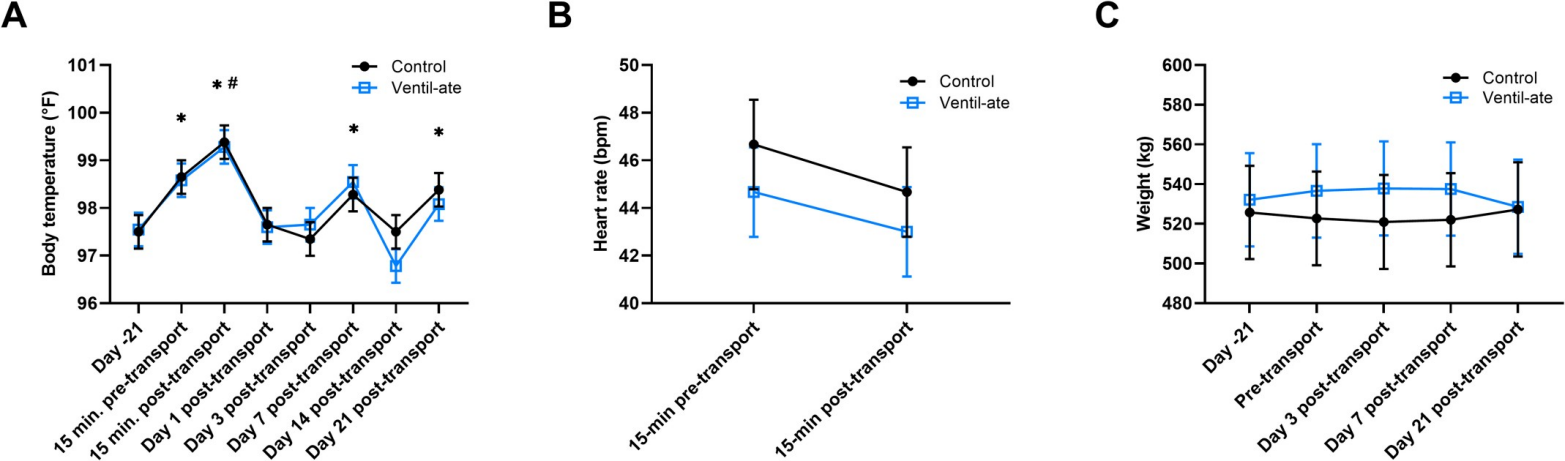
(A) Body temperature, (B) heart rate, and (C) body weight. For illustrative purposes, the results (least squares means ± SE) shown are from the analyses that included the group-by-time interaction; however, the significant differences noted on the figure were determined from the analysis with only group and time included as main effects. (A) No significant group (*P* = 0.9105) or group-by-time (*P* = 0.8787) effects were observed; a significant time effect was observed (*P* < 0.0001). * denotes a significant difference (*P* < 0.05) from baseline (Day –21). ^#^ denotes a significant difference (*P* < 0.001) from all other time points. (B) No significant group (*P* = 0.4316), time (*P* = 0.2043), or group-by-time (*P* = 0.9091) effects were observed. Bpm, beats per minute. (C) No significant group (*P* = 0.8464), time (*P* = 0.9878), or group-by-time (*P* = 0.4057) effects were observed.

No significant group (*P* = 0.9105) or group-by-time (*P* = 0.8787) effects were observed in the analysis of body temperature, but there was a significant time effect (*P* < 0.0001; Fig 1A). Body temperature was increased compared to baseline (Day –21) values at 15-min pre-transport (*P* < 0.0001), 15-min post-transport (*P* < 0.0001), Day 7 (*P* < 0.0001), and Day 21 (*P* = 0.0172), and body temperature at 15-min post-transport was also increased compared to all other timepoints (*P*-value range: < 0.0001–0.0004). One value for body temperature on Day 14 was quite low (94.2°F); therefore, the data were also analyzed with this value excluded. In this analysis, no significant group (*P* = 0.9322) or group-by-time (*P* = 0.9852) effects were observed, but the time effect was significant (*P* < 0.0001). Body temperature was increased compared to baseline values at 15-min pre-transport (*P* = 0.0004), 15-min post-transport (*P* < 0.0001), Day 7 (*P* = 0.0063), and Day 21 (*P* = 0.0285), and body temperature at 15-min post-transport was also increased compared to all other timepoints (*P*-value range: < 0.0001–0.017). Thus, removal of this value did not affect the interpretation of the results. No significant group, time, or group-by-time effects were observed in the analyses of heart rate (*P* = 0.4316, *P* = 0.2043, *P* = 0.9091, respectively; Fig 1B) or body weight (*P* = 0.8464, *P* = 0.9878, *P* = 0.4057, respectively; Fig 1C).

### Serum total cortisol

**Fig 2:**
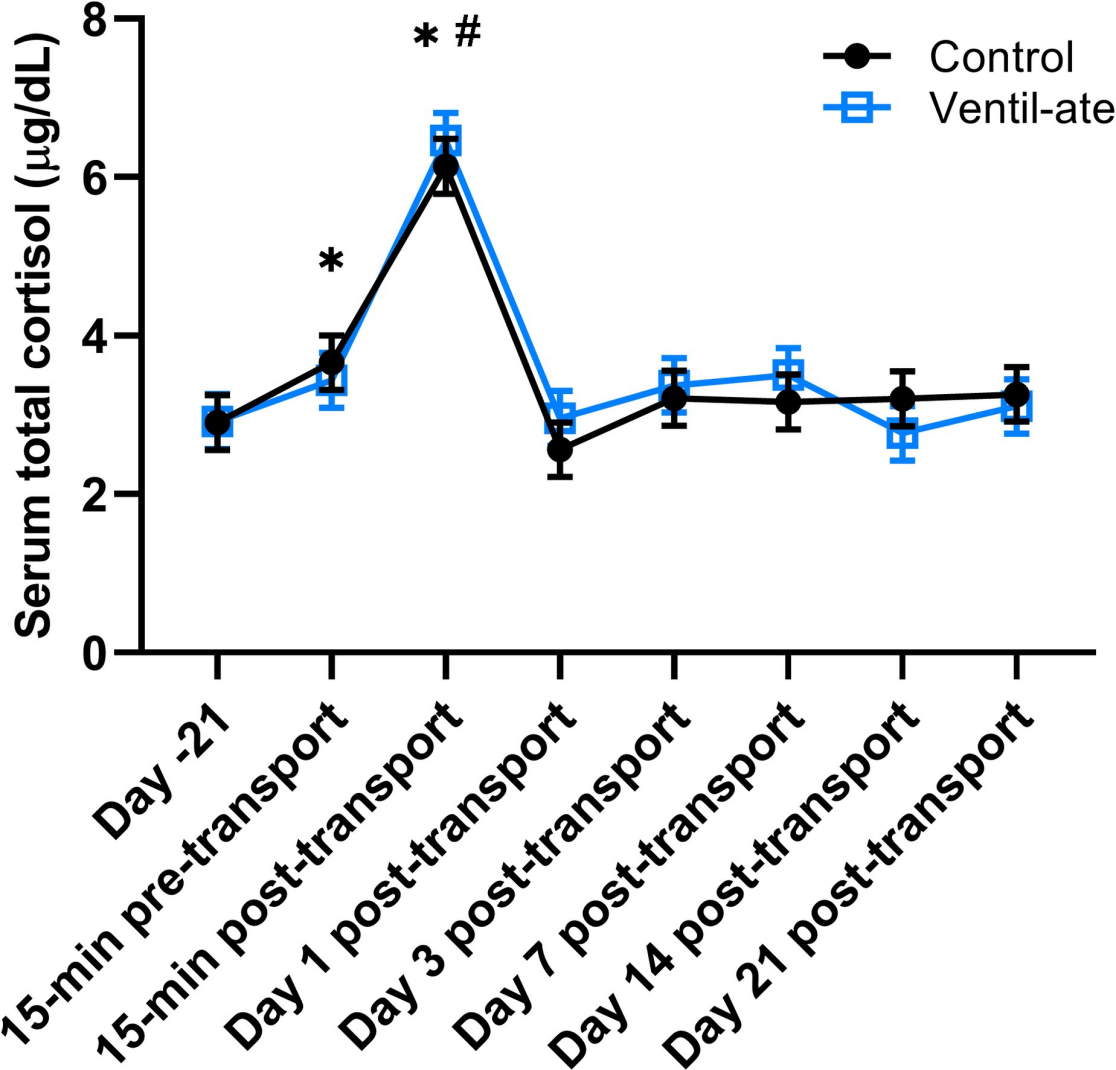
Serum total cortisol concentration. For illustrative purposes, the figure reflects the results (least squares means ± SE) from the analysis that included the group-by-time interaction; however, the significant time differences noted on the figure reflect the results of the analysis with only group and time included as main effects. No significant group (*P* = 0.8804) or group-by-time (*P* = 0.7053) effects were observed, but there was a significant time effect (*P* < 0.0001). * denotes a significant difference (*P* < 0.05) from baseline (Day –21). ^#^ denotes a significant difference (*P* < 0.0001) from all other time points.

No significant group (*P* = 0.8804) or group-by-time (*P* = 0.7053) effects were observed, but there was a significant time effect (*P* < 0.0001; Fig 2). Total cortisol concentrations at 15-min pre- and post-transport were increased compared with baseline (Day –21) values (*P* = 0.0165 and *P* < 0.0001, respectively). In addition, total cortisol concentrations were significantly higher at 15-min post-transport compared to all other time points (*P* < 0.0001 for all comparisons).

Total cortisol concentrations were also analyzed after natural log transformation, and the same pattern of results was observed. The group (*P* = 0.9869) and group-by-time (*P* = 0.3456) effects were not significant, but there was a significant time effect (*P* < 0.0001). Total cortisol concentrations at 15-min pre- and post-transport were increased compared with baseline (Day –21) values (*P* = 0.0318 and *P* < 0.0001, respectively), and total cortisol concentrations at 15-min post-transport were significantly higher than all other time points (*P* < 0.0001 for all comparisons).

### Gene expression in whole blood

**Fig 3:**
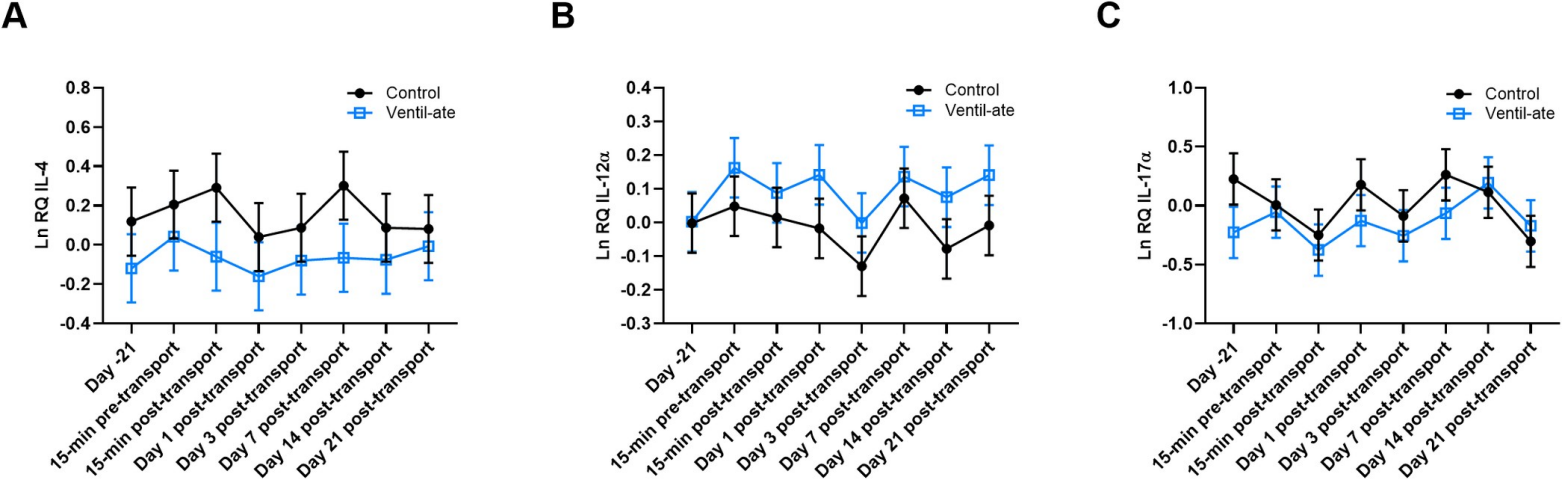
Whole blood gene expression of (A) IL-4, (B) IL-12α with baseline (Day –21) values included as a covariate, and (C) IL-17α with baseline (Day –21) values included as a covariate. Relative quantities (RQ) are natural log (Ln) transformed. For illustrative purposes, the figure displays the results (least squares means ± SE) from the analyses that included the group-by-time interaction. (A–C) No significant group, time, or group-by-time effects were observed. IL, interleukin.

Initial analyses revealed baseline (Day –21) differences between the control and supplemented groups for LnIL-2, LnIL-10, LnIL-12α, LnIL-17α, LnIFNγ, and LnTNFα; therefore, the respective baseline values were included as covariates in these analyses. No significant group-by-time effects were observed for LnIL-1β (*P* = 0.2555), LnIL-4 (*P* = 0.6898), LnIL-6 (*P* = 0.4411), LnIL-8 (*P* = 0.943), and LnSAA1 (*P* = 0.9067). Furthermore, no significant group-by-time effects were observed for LnIL-2, LnIL-10, LnIL-12α, LnIL-17α, LnIFNγ, or LnTNFα, either with baseline (Day –21) values included as covariates (*P* = 0.5933, 0.4287, 0.8854, 0.8296, 0.7791, and 0.8095, respectively) or without (*P* = 0.5656, 0.4304, 0.8854, 0.8507, 0.7791, and 0.8101, respectively). In addition, no significant group (*P* = 0.3487) or time (*P* = 0.2153) effects were observed in the analysis of LnIL-4 expression (Fig 3A). There were also no significant group or time effects for LnIL-12α and LnIL-17α, either with baseline values included as covariates [LnIL-12α: *P* = 0.3715 (group), *P* = 0.0554 (time); LnIL-17α: *P* = 0.2013 (group), *P* = 0.2581 (time)] or without [LnIL-12α: *P* = 0.0504 (group), *P* = 0.0554 (time); LnIL-17α: *P* = 0.074 (group), *P* = 0.2526 (time); Fig 3B and 3C].

**Fig 4:**
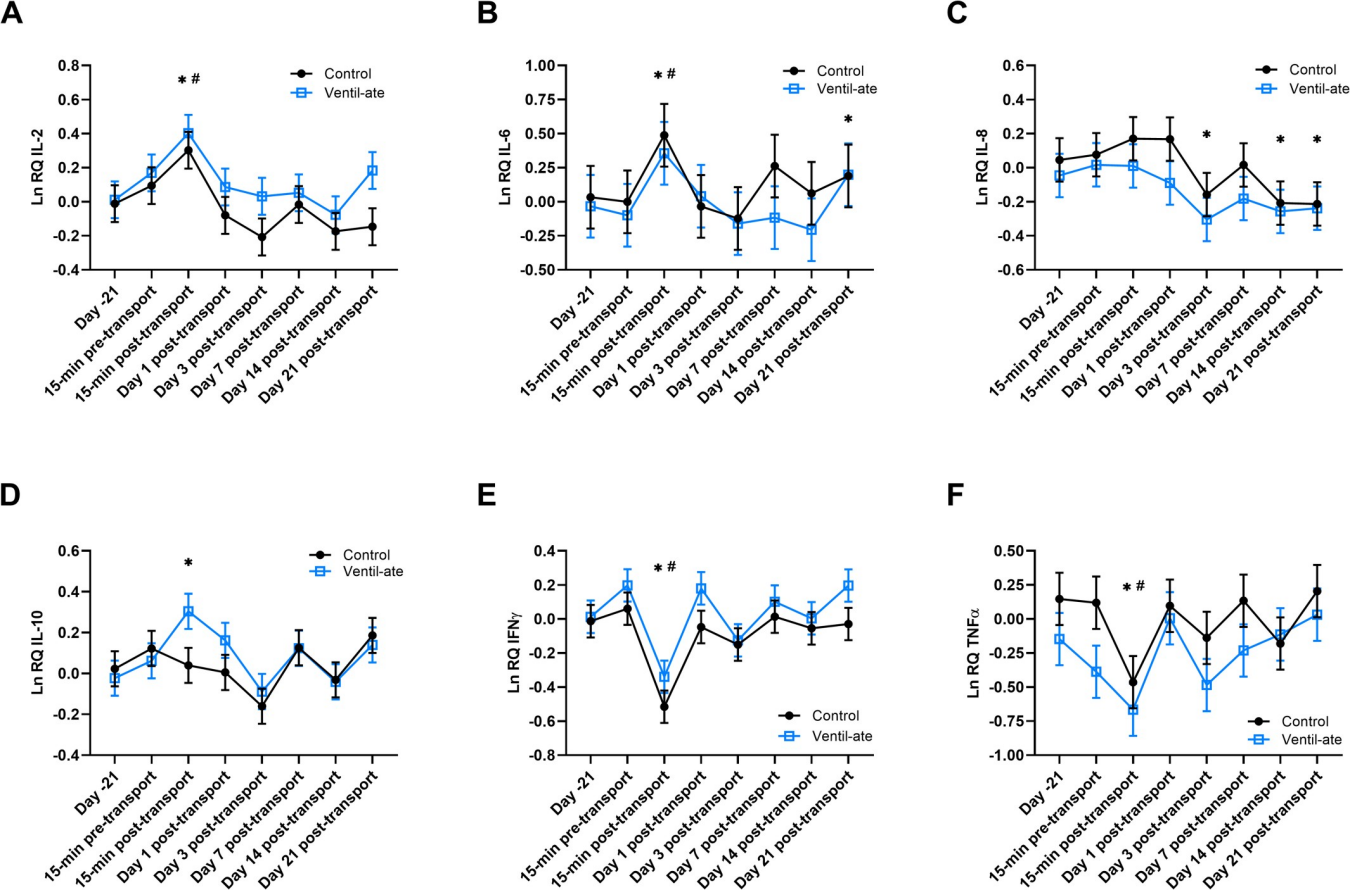
Whole blood gene expression of (A) IL-2 with baseline (Day –21) values included as a covariate, (B) IL-6, (C) IL-8, (D) IL-10 with baseline (Day –21) values included as a covariate, (E) IFNγ with baseline (Day –21) values included as a covariate, and (F) TNFα with baseline (Day –21) values included as a covariate. Relative quantities (RQ) are natural log (Ln) transformed. For illustrative purposes, the figure displays the results (least squares means ± SE) from the analyses that included the group-by-time interaction; however, the significant differences noted on the figures were determined from the analyses with only group and time included as main effects. (A–F) No significant group or group-by-time effects were observed, but significant time effects were detected (A, B, E: *P* < 0.0001 and C, D, F: *P* < 0.01). * denotes a significant difference (*P* < 0.05) from baseline (Day –21). (A, B, E) ^#^ denotes a significant difference (*P* < 0.05) from all other time points. (F) ^#^ denotes a significant difference (*P* < 0.05) from all other time points except Day 3 post-transport. IFNγ, interferon gamma; IL, interleukin; TNFα, tumor necrosis factor alpha.

Significant time effects were observed for LnIL-2 (*P* < 0.0001), LnIL-6 (*P* < 0.0001), LnIL-8 (*P* = 0.0035), LnIL-10 [with (*P* = 0.0076) and without (*P* = 0.0045) baseline values as a covariate], LnIFNγ (*P* < 0.0001), and LnTNFα [with (*P* = 0.003) and without (*P* = 0.0031) baseline values as a covariate; Fig 4A–4F]. There were no significant group effects in the analyses of LnIL-6 (*P* = 0.1026), LnIL-8 (*P* = 0.3636), LnIL-2 [with (*P* = 0.282) and without (*P* = 0.1514) baseline values as a covariate] or LnIFNγ [with (*P* = 0.2539) and without (*P* = 0.0536) baseline values as a covariate] expression. Significant group effects were initially observed for LnIL-10 (*P* = 0.0173) and LnTNFα (*P* = 0.0112) but not when baseline values were included as a covariate (*P* = 0.4916 and *P* = 0.173, respectively).

When baseline values were included as a covariate, LnIL-2 expression was increased at 15-min post-transport compared to baseline (Day –21; *P* < 0.0001) and all other timepoints (*P*-value range: < 0.0001–0.0054). Expression of LnIL-6 was also increased compared to baseline (Day –21; *P* < 0.0001) and all other timepoints (*P*-value range: < 0.0001–0.048). Interestingly, LnIL-6 expression was also increased compared with baseline (Day –21) values at Day 21 post-transport (*P* = 0.0056). In contrast, LnIFNγ expression at 15-min post-transport was decreased compared to baseline (*P* < 0.0001) and all other timepoints (*P*-value range: < 0.0001–0.0003) regardless of whether baseline values were included as a covariate. Expression of LnTNFα was also decreased at 15-min post-transport compared with baseline (Day –21; *P* = 0.0012) and all other timepoints (*P*-value range: 0.0002–0.0165) except for Day 3 post-transport (*P* = 0.1369) when baseline values were included as a covariate. By Day 1 post-transport, LnIL-2, LnIL-6, LnIFNγ, and LnTNFα expression had returned to baseline (Day –21) levels.

On Days 3, 14, and 21 post-transport, LnIL-8 expression was significantly lower than baseline (Day –21) levels (*P* = 0.0229, *P* = 0.026, and *P* = 0.0325, respectively). When baseline values were included as a covariate, LnIL-10 expression was increased at 15-min post-transport compared to baseline values (*P* = 0.0423). The significant increase in LnIL-10 expression at 15-min post-transport visually appeared to be driven by changes in the supplementation group; however, given that a significant group-by-time effect was not observed, the time effects for each group were not examined separately.

**Fig 5:**
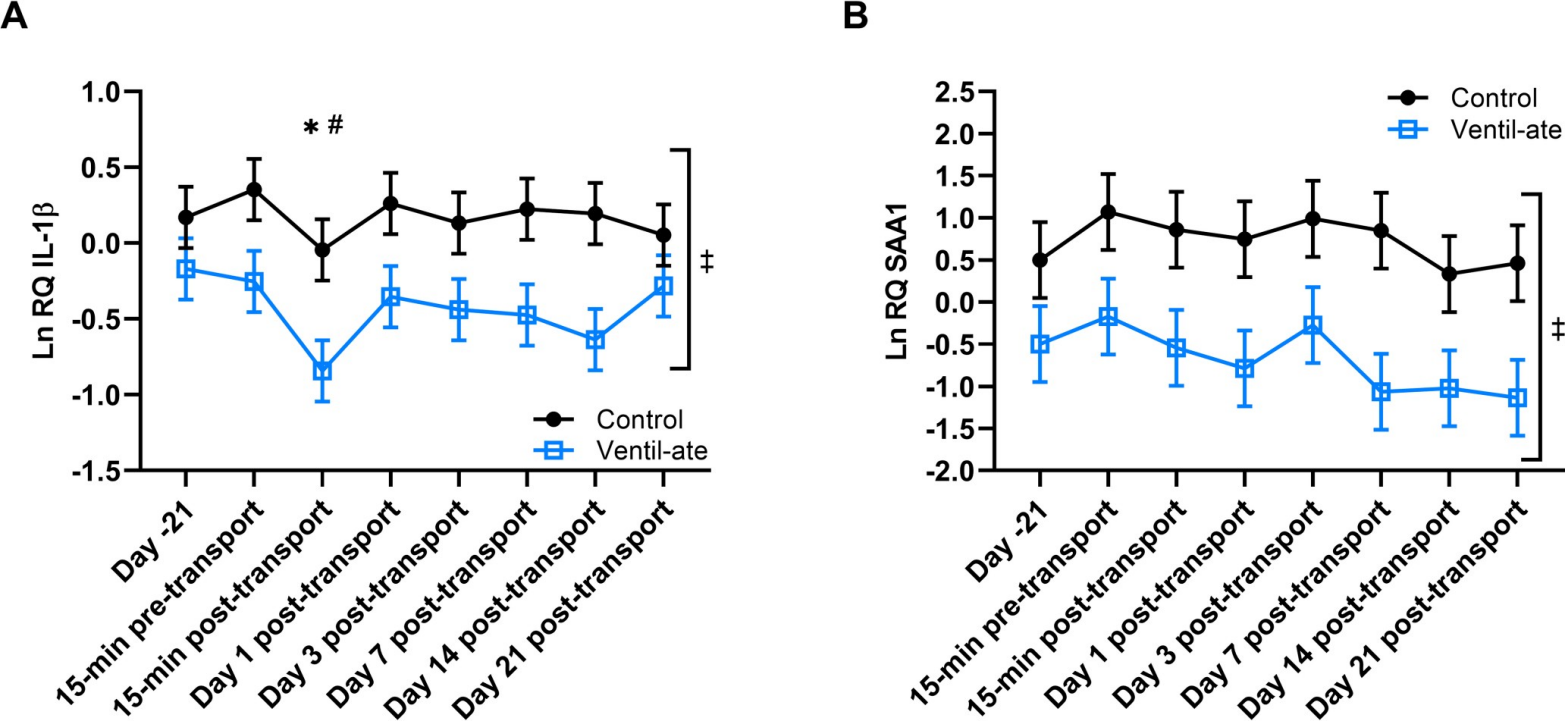
Expression of (A) IL-1β and (B) SAA1 in whole blood. Relative quantities (RQ) are natural log (Ln) transformed. For illustrative purposes, the figure displays results (least squares means ± SE) from the analyses that included the group-by-time interaction; however, the significant differences shown were determined from the analyses with only group and time included as main effects. (A) Significant group (*P* = 0.037) and time (*P* = 0.0025) effects were observed; the group-by-time effect was not significant (*P* = 0.2555). * denotes a significant difference (*P* < 0.05) from baseline (Day –21). ^#^ denotes a significant difference (*P* < 0.05) from all time points except Day 14 post-transport. (B) A significant group effect was detected (*P* = 0.0183); the time (*P* = 0.1245) and group-by-time (*P* = 0.9067) effects were not significant. ^‡^ denotes the significant overall group difference (*P* = 0.0183) between the control and supplemented groups. IL, interleukin; SAA, serum amyloid A.

Significant group (*P* = 0.037) and time (*P* = 0.0025) effects were detected in the analysis of LnIL-1β expression (Fig 5A). Only a significant group effect (*P* = 0.0183) was detected in the analysis of LnSAA1 expression; the time effect (*P* = 0.1245) was not significant (Fig 5B). The supplemented group generally had lower LnIL-1β and LnSAA1 expression than the control group. At 15-min post-transport, LnIL-1β expression was decreased compared to baseline (Day –21; *P* = 0.0003) and to all other timepoints (*P*-value range: < 0.0001–0.0164) except Day 14 post-transport (*P* = 0.0789). Therefore, while group status (control versus supplemented) did not appear to alter the expression of LnIL-1β and LnSAA1 in response to transport, supplementation appeared to generally reduce LnIL-1β and LnSAA1 expression.

### PBMCs: Flow cytometry

**Fig 6:**
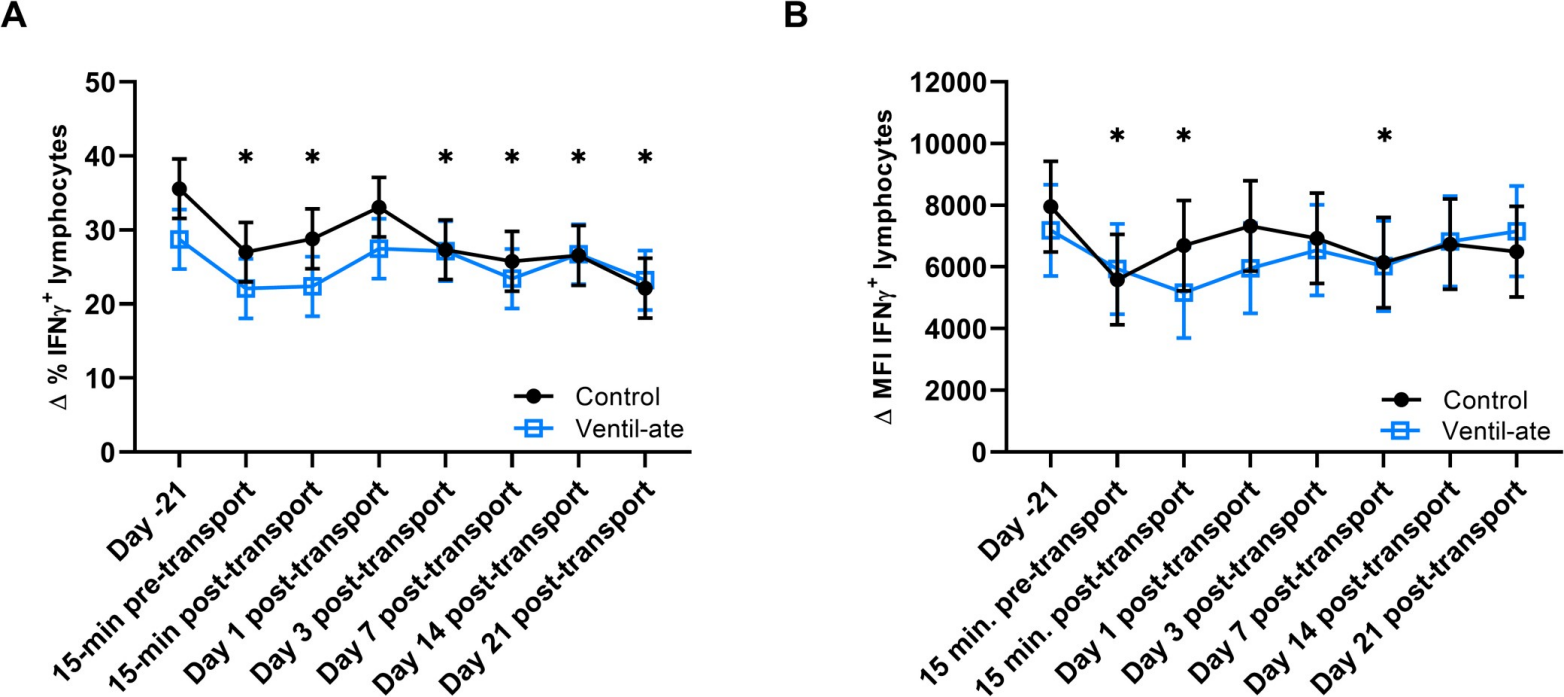
Change (Δ) in the (A) percentage (%) and (B) mean fluorescence intensity (MFI; geometric mean) of IFNγ^+^ lymphocytes after stimulation and with media values included as a covariate. For illustrative purposes, the figure displays results (least squares means ± SE) from the analyses that included the group-by-time interaction; however, the significant differences shown were determined from the analyses with only group and time included as main effects. (A–B) Significant time effects were observed (*P* = 0.0016 and *P* = 0.0039, respectively). * denotes a significant difference from baseline (Day –21). IFNγ, interferon gamma.

For the Δ % of IFNγ^+^ lymphocytes after stimulation, the group (*P* = 0.5333) and group-by-time (*P* = 0.5464) effects were not significant, but a significant time effect (*P* = 0.0016) was observed. The Δ % of IFNγ^+^ lymphocytes after stimulation was decreased compared with baseline (Day –21) values at all time points (*P*-value range: 0.0005–0.0426) except Day 1 post-transport (*P* = 0.4184; Fig 6A). For the Δ MFI of IFNγ^+^ lymphocytes after stimulation, the group (*P* = 0.8827) and group-by-time (*P* = 0.251) effects were not significant, but a significant time effect was also observed (*P* = 0.0039). Compared with baseline (Day –21) values, the Δ MFI of IFNγ^+^ lymphocytes after stimulation was decreased at the 15-min pre-transport (*P* = 0.0002), 15-min post-transport (*P* = 0.001), and Day 7 post-transport (*P* = 0.0053) time points (Fig 6B).

**Fig 7:**
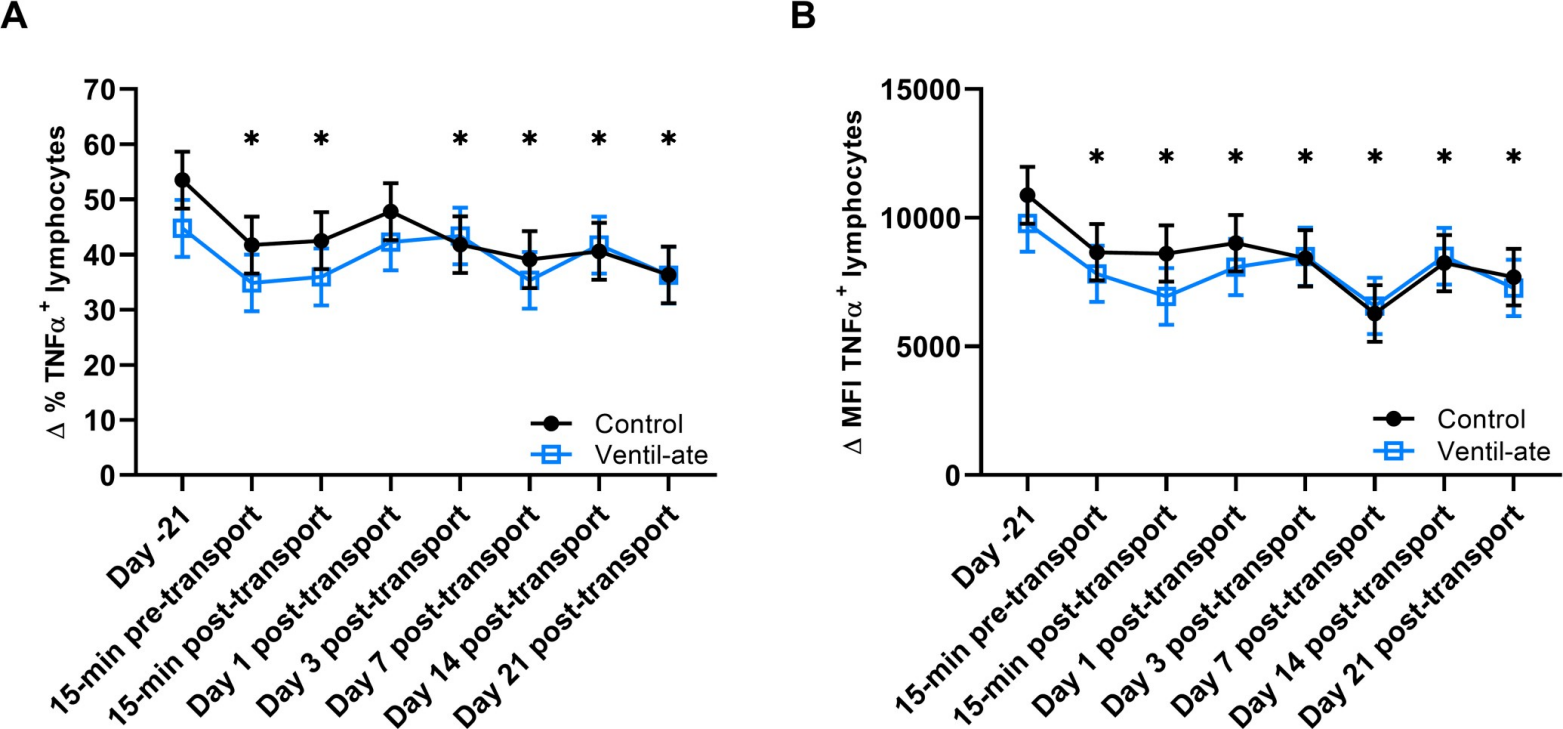
Change (Δ) in the (A) percentage (%) and (B) mean fluorescence intensity (MFI; geometric mean) of TNFα^+^ lymphocytes after stimulation and with media values included as a covariate. For illustrative purposes, the figure displays results (least squares means ± SE) from the analyses that included the group-by-time interaction; however, the significant differences shown were determined from the analyses with only group and time included as main effects. (A–B) Significant time effects were observed (*P* = 0.0004 and *P* < 0.0001, respectively). * denotes a significant difference from baseline (Day –21). TNFα, tumor necrosis factor alpha.

For the Δ % of TNFα ^+^ lymphocytes after stimulation, the group (*P* = 0.5572) and group-by-time (*P* = 0.4873) effects were not significant; however, a significant time effect was observed (*P* = 0.0004). Compared with baseline (Day –21) values, the Δ % of TNFα ^+^ lymphocytes after stimulation was reduced at all time points (*P*-value range: 0.0001–0.0317) except Day 1 post-transport (*P* = 0.1637; Fig 7A). For the Δ MFI of TNFα ^+^ lymphocytes after stimulation, a significant time effect was also observed (*P* < 0.0001), but the group (*P* = 0.6676) and group-by-time (*P* = 0.8161) effects were not significant. The Δ MFI of TNFα ^+^ lymphocytes after stimulation was significantly reduced at all time points (*P*-value range: < 0.0001–0.0103) compared with baseline (Day –21) values (Fig 7B).

### Correlations at 15-min post-transport

Age did not correlate with any of the measures. All significant correlations are shown in Table 1.

**Table 1:** Significant correlations at 15-min post-transport only.

Variable	Correlated with:	R; *P*-value
Δ % of TNFα^+^ lymphocytes	Δ MFI of TNFα^+^ lymphocytes	0.91836; < 0.0001
	Δ % of IFNγ^+^ lymphocytes	0.91478; < 0.0001
	LnIL-12α	0.71295; 0.0092
	LnTNFα	0.62577; 0.0295
Δ MFI of TNFα^+^ lymphocytes	Δ % of IFNγ^+^ lymphocytes	0.88585; 0.0001
	LnIL-12α	0.73058; 0.007
	LnTNFα	0.60256; 0.0381
Δ % of IFNγ^+^ lymphocytes	LnIL-12α	0.71976; 0.0083
	LnTNFα	0.63268; 0.0272
LnIL-12α	LnIFNγ	0.62014; 0.0315
	LnIL-2	0.73773; 0.0062
	LnIL-6	0.71065; 0.0096
	LnIL-10	0.73794; 0.0061
	LnTNFα	0.92475; < 0.0001
LnTNFα	LnIL-2	0.78448; 0.0025
	LnIL-6	0.68807; 0.0134
	LnIL-10	0.85973; 0.0003
LnIL-2	LnIL-4	0.73522; 0.0064
	LnIL-6	0.6516; 0.0217
	LnIL-10	0.75008; 0.005
LnIL-10	LnIL-4	0.58133; 0.0474
	LnIL-6	0.8453; 0.0005
LnIL-1β	LnSAA1	0.91226; < 0.0001
LnIL-17α	Body temperature	0.6129; 0.0341

^a^Abbreviations: IFNγ, interferon gamma; IL, interleukin; Ln, natural log; MFI, mean fluorescence intensity (geometric mean); SAA, serum amyloid A; TNFα, tumor necrosis factor alpha.

## Discussion

Activation of the HPA axis and sympathetic nervous system is generally the first reaction to acute stress. Current research on the effects of transport as a stressor in horses has primarily focused on long-distance transport (i.e., ≥ 24 hr duration), particularly in studies where immune responses were evaluated [9,13,14]. However, several studies in horses have demonstrated that shorter transport times (i.e., ≤ 8 hr) also induce acute stress and activate the HPA axis [15,16,27]. In one study, horses had increased ACTH and cortisol concentrations after being transported 100 km and 200 km (between 1 and 3 hr), and another found that salivary cortisol increased after only one hour of transport [15,16]. Other research has demonstrated that even well-behaved horses have increases in heart rate just from the act of getting on the trailer, while horses that were not “easy-loaders” had increased cortisol and heart rates from the loading process [28]. With many horse owners transporting their horses across short distances for sporting, leisure, and breeding activities, it is important to further understand the effects of transport stress on immune function, particularly for shorter transport times.

As mentioned, there is a growing body of research demonstrating that long-distance transport of horses leads to altered immune function, and that longer transport times (24 hr– 49 hr) increase the risk of illness [3,9,13,14]. In horses transported for longer periods of time, increases in WBC and neutrophil counts have been observed, and Stull et al (2004) also found that lymphocyte subpopulations were decreased in horses traveling for 24 hr but recovered within 24 hr of transport completion [7,9,13,14,29–31]. In 2008, Stull et al also compared transport times of 24 hr (i.e., all at once) to two 12-hr trips separated by a 12-hr rest period; they found similar results to the 24 hr trip, except that CD_3_^+^, CD_4_^+^, and CD_8_b^+^ populations improved after the transport time was split with rest time incorporated [13,14]. Research has also shown that the onset of clinical signs associated with shipping-related illness generally occurs within the first 20–48 hr of transport [1,3,30]. Together, these findings strongly suggest that there is an early window during which transport stress affects immune function. Indeed, the results of the current study are consistent with this conclusion, because the observed changes associated with transport had often normalized at or before the Day 1 sample collections.

Because immune responses have the potential to be both protective and destructive, they are highly regulated in healthy individuals. A strong pro-inflammatory response is generally considered protective during acute illness or stress, but an extended pro-inflammatory response can be damaging and must cease when no longer needed. One of the earliest immune responses during illness and stress is the acute phase response, which is a rapid, non-specific defense mechanism. After HPA axis activation and cortisol release, expression of TNFα, IL-1β, and IFNγ typically increases and induces the acute phase response, which includes the production of acute phase proteins such as serum amyloid A [32].

In this study, supplementation significantly reduced overall IL-1β and SAA1 expression. Reductions in both IL-1β and SAA1 expression are consistent with the anti-inflammatory properties of the supplement used [33]. These results also indicate reduced inflammation associated with the acute phase response, which is generally expected to be stimulated by acute stress, such as transport. IL-1β and SAA are closely connected in their roles in the acute phase response; therefore, the agreement of the results strengthens this conclusion [33].

Interestingly though, TNFα, IL-1β, and IFNγ expression in whole blood were decreased at 15-min post-transport, unlike SAA1, which suggests that the acute phase response was not activated by transport stress as would be expected [34]. However, total cortisol and body temperature were significantly increased at this time point; these are normal responses to HPA activation and confirm that acute stress was induced by transport and that the HPA axis was activated [32]. The decreased expression of TNFα, IL-1β, and IFNγ is also inconsistent with research in other species. In one recent study, Li et al (2019) showed that TNFα, IL-1β, and IL-6 were elevated in cattle transported for 6 hr [17]. Thus, it is potentially concerning that TNFα, IL-1β, and IFNγ were decreased as a result of transport in this study. One possible explanation for these results is that initial responses to cortisol, which was also significantly higher than baseline at 15-min pre-transport, triggered earlier changes that were missed during the window between the 15-min pre- and post-transport sample collections. Thus, these results may reflect “rebound” changes that were occurring during an attempt to maintain homeostasis in the immune response. Another possible explanation is that the decreased responses of these pro-inflammatory cytokines to stress are the result of age, although age did not correlate with any of the measures at 15-min post-transport. Declines in immune function are well-documented in aging horses, but transport studies in horses and other species tend to include younger animals rather than aged animals [22,35–40].

Unfortunately, both of these explanations (“rebound effect” versus age) are potentially concerning. In either case, horses appear to have a potentially vulnerable window during or shortly after transport in which acute, pro-inflammatory immune responses are dampened. This, along with other transport factors such as head position, ventilation, etc., may be sufficient to allow for the onset of bacterial infection, reactivation of latent viral infection, or for other infections associated with new environments and exposures to gain an advantage [9,10,12,30,41].

Additionally, expression of IL-2 and IL-6, which are also considered pro-inflammatory cytokines, as well as IL-10, which is generally considered an anti-inflammatory cytokine, were significantly increased at 15-min post-transport. Though there was not a significant group-by-time interaction, the supplemented group visually appeared to have a larger increase in IL-10 expression than the control horses. However, IL-4, another anti-inflammatory cytokine, was not impacted by transport. If the “rebound” effects discussed earlier were already in process at the 15-min post-transport time point, it would have been expected that both IL-4 and IL-10 would also have been elevated in all horses. These results suggest that the “rebound effect” may not fully explain the observed findings from this study.

Compared to baseline (Day –21), both the control and supplemented groups also had decreases in the changes (Δ) in the percentage and MFI of IFNγ^+^ and TNFα^+^ lymphocytes in response to stimulation at numerous timepoints. However, unlike the significant time effects seen in the analyses of whole blood gene expression, the observed changes were not primarily observed at the time points immediately before and after transport. Thus, these changes may indicate early activation of the stress response, potentially during the handling process (i.e., 15-min pre-transport), effects of transport stress that extended beyond the 21 days in which post-transport immune function was assessed in this study, seasonal changes in immune function, or elevated baseline values (due to an unknown reason) with the remaining time points reflecting normal immune responses, as well as a combination of one or more of these potential explanations [21]. Though it is not possible to determine a cause based on this study alone, the changes in the percentage and MFI of IFNγ^+^ and TNFα^+^ lymphocytes in response to stimulation may not be adequately explained solely by the effects of transport stress, suggesting that there may be other factors that also contributed to the observed results.

In addition to the limited sample size, one of the limitations of this study was that the stress response was initiated prior to travel (15-min pre-transport), as indicated by the significant increases in total cortisol and body temperature at this time point. The similarities in heart rate at 15-min pre- and post-transport further support the conclusion that the stress response had already been initiated prior to transport. This may have resulted from the horses’ attention to a slight change in their daily schedules, particularly regarding feeding times. Thus, the true peak in total cortisol likely occurred at some point between the 15-min pre- and post-transport time points; increased sample collections during this time period would be necessary for future work. Additionally, it is possible that aged horses respond differently to short-term transport stress than young or adult horses. Without having a young group of horses in this study, it is not possible to determine whether the observed effects of short-term transport or supplementation are applicable to horses of all ages or are unique to aged horses. Future research in this area would benefit from having both young and aged horses in order to make these comparisons. It was also not possible to evaluate the effects of sex on the measured responses to transport or supplementation, because the horses were assigned to the control and treatment groups based on age and Day –35 cytokine production by lymphocytes. Thus, given the limited number of horses in the study, only one gelding was assigned to the supplementation group. While the authors recognize several limitations in the present study, this work is important in that the findings of altered immune function are consistent with long-distance transport studies and provide valuable information for the design of future studies.

Overall, the results of this study demonstrate that short-term transport significantly affects numerous aspects of equine immune function, particularly within the first 24 hr of transport, and may reflect altered activation of the acute phase and pro-inflammatory responses to transport stress. This is potentially concerning given both the frequency with which horses are transported across short distances as well as the consistency of the results with other research findings that have demonstrated that immune function is affected by long-term transportation. In this study, supplementation also decreased IL-1β and SAA1 expression, which was consistent with the supplement’s anti-inflammatory properties [19,20]. These findings demonstrate that supplementation can successfully alter inflammation in horses and could potentially be used to modulate the immune response.

Considering the findings in this study and in other published work, further research into the immunological effects of transport stress in horses is warranted, especially for shorter transport durations and distances. Future work should focus on the first 24 hr after transport initiation to determine how immune function responds during this window. Additionally, investigations of the effects of age and trip frequency are also necessary to better understand the potential risks that may accompany transport. This study indicates that older horses being transported for a short distance and/or duration may have altered immune responses, which could increase their susceptibility to transport-related illnesses. Although further work is needed to confirm this, additional caution should be taken in the meantime when transporting these horses, such as by ensuring adherence to general biosecurity precautions and including increased monitoring for any potential adverse effects.

## Supporting information

S1 File Study dataset. (XLSX)
